# Cardiac arrest in the Australian Alps: A 20-year analysis

**DOI:** 10.1016/j.hroo.2025.03.004

**Published:** 2025-03-14

**Authors:** Elizabeth D. Paratz, Emily Nehme, Ashanti Dantanarayana, Kelila Freedman, Daniel Coakley, Louise Fahy, Stephanie Rowe, Bruce Wilkie, Adam Trytell, David Anderson, Andreas Pflaumer, Dion Stub, Andre La Gerche, Ziad Nehme

**Affiliations:** 1 Centre for Research and Evaluation, Ambulance Victoria, Blackburn, Australia; 2 HEART Lab, St Vincent’s Institute of Medical Research, Fitzroy, Australia; 3 HEART Lab, Victor Chang Cardiac Research Institute, Darlinghurst, Australia; 4 Department of Cardiology, St Vincent’s Hospital Melbourne, Fitzroy, Australia; 5 Faculty of Medicine, Dentistry and Health Sciences, University of Melbourne, Fitzroy, Australia; 6 Department of Cardiology, Alfred Health, Prahran, Australia; 7 Department of Surgery, Eastern Health, Box Hill, Australia; 8 Department of Cardiology, Royal Children’s Hospital, Melbourne, Australia; 9 Department of Paediatrics, University of Melbourne, Parkville, Australia; 10 Department of Cardiology, Murdoch Children’s Research Institute, Parkville, Australia

**Keywords:** Alpine environment, Snow, Cardiac arrest, Resuscitation, Cardiology

## Abstract

**Background:**

Alpine tourism annually attracts over 100 million visitors globally. Age and cardiovascular comorbidities in alpine tourists are increasing, and rates of out-of-hospital cardiac arrest (OHCA) have been hypothesized to be higher due to exertion and physiological stress.

**Methods:**

Cases of alpine OHCA from 2002 to 2021 were identified from the statewide Victorian Ambulance Cardiac Arrest Registry. Alpine and nonalpine OHCA characteristics were compared. Causes of alpine OHCA were obtained from hospital discharge diagnoses and the National Coronial Information System.

**Results:**

Approximately 15.3 million alpine visits were recorded over the time period, during which 13 alpine OHCAs occurred (0.04% of 32,179 OHCAs, 0.8 OHCAs per million alpine visits). Compared with nonalpine OHCAs in a public setting, alpine OHCA patients were younger (median age 52 years vs 63 years, *P =* .0373), with higher rates of bystander defibrillation (54.5% vs 13.5%, *P <* .0001). Survival to hospital discharge did not significantly differ between alpine (38.5%) and nonalpine OHCA patients. Ischemic heart disease was the commonest identified cause of alpine OHCA in both survivors and nonsurvivors.

**Conclusion:**

Alpine OHCA is very rare in Australia, accounting for 1 in 5000 OHCAs and fewer than 1 in a million ski field visitors. Despite remoteness and access challenges, alpine OHCA survival is high, driven by prognostically favorable arrest-related factors and coordinated local systems of care prioritizing early bystander intervention.

## Graphical abstract

**Figure:**
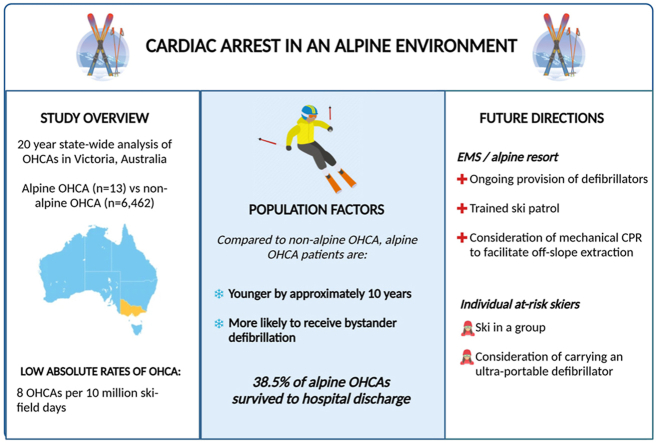

**Key Findings**

▪ Alpine out-of-hospital cardiac arrest (OHCA) is very rare in Australia, accounting for 1 in 5000 OHCAs and fewer than 1 in a million ski field visitors.
▪ Despite remoteness and access challenges, alpine OHCA survival is high, at 38.5%. This may relate to younger age and higher rates of bystander defibrillation compared with nonalpine OHCA in public places.
▪ Ongoing efforts to support early bystander response and training of ski patrol remain very important.

## Introduction

Downhill skiing is the world’s most popular winter mountain sport, and there are approximately 100 million alpine tourist visits globally each year, equating to 400 million downhill skiing and snowboarding days.^1^^,^^2^ The “Australian Alps,” which span the 2 states of Victoria and New South Wales, typically receive approximately 2 million annual visitors.^3^

Concerns have previously been raised that exertion in the snow, with superimposed cold and altitude stress, may increase the risk of myocardial infarction or cardiac arrest,^4^ with cardiac arrest the most common cause of death in the mountains.^2^^,^^5^ In 2006, Davies and colleagues^6^ stated that “downhill skiing … is considered to be a serious trigger for sudden cardiac deaths in those with previous myocardial infarction, and also in those with hypertension, known coronary heart disease, or with poor adaptation to strenuous exercise,” and in 2007 Burtscher^7^ quantified a 93-fold risk of sudden cardiac arrest (SCA) for skiers who had experienced previous myocardial infarction. In a Finnish study, skiing was the commonest trigger of SCA during exertion.^8^

The median age and prevalence of alpine visitors with cardiovascular comorbidities is increasing.^1^^,^^9^ Emergency medical services (EMS) response time and patient retrieval may also be challenging in the alpine environment, influenced by remoteness, altitude, and unpredictable weather conditions.^1^ As such, alpine out-of-hospital cardiac arrests (OHCAs) present a unique subset of OHCA with possible variations in incidence, patient demographics and resuscitation environment. Examining this unique environment and tailoring local systems of care to enhance OHCA survival is important, particularly given the international popularity of alpine sports.

This study utilized twenty years of data from the state-wide Victorian OHCA registry to assess presentation and outcomes in alpine OHCA, with linkage to hospital discharge diagnoses and a national coronial system.

## Methods

### Data sources

The Victorian Ambulance Cardiac Arrest Registry (VACAR) records data on all OHCAs attended by EMS within the state of Victoria, Australia (population 6.6 million). Its methods have been described in detail previously, with all patient-level details collected according to standard Utstein template metrics.^10^ The VACAR contains full geocoding details, permitting identification of patient location at time of OHCA. Missingness of data is very low within the registry, at <1% across all variables.^11^ The VACAR receives hospital discharge diagnoses routinely for all patients transported to hospital as part of standard data linkage processes. Forensic data (postmortem, police narratives of circumstances, and toxicology reports) were obtained from the National Coronial Information System (NCIS).

### Alpine environment

**Figure 1:**
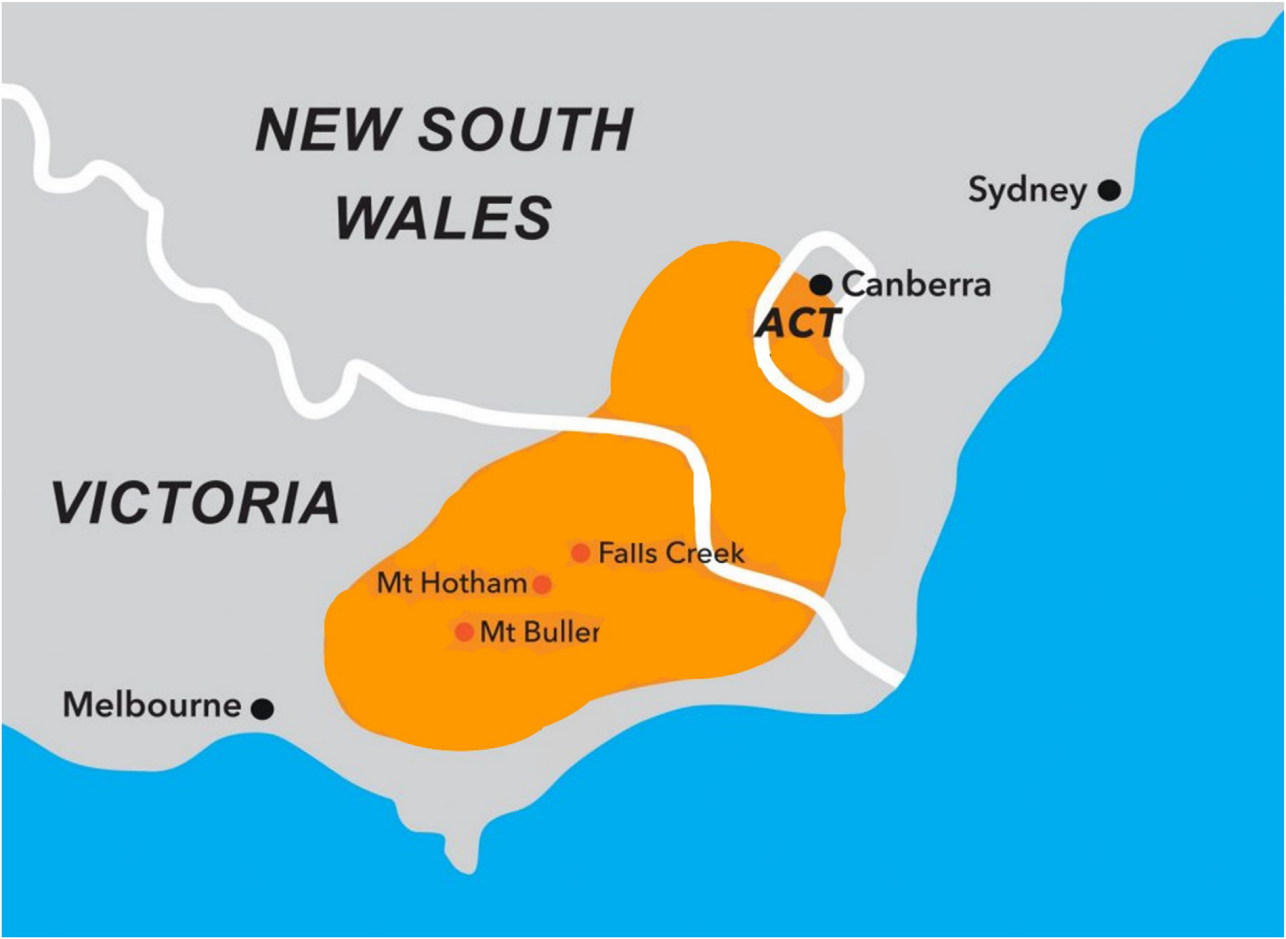
The Australian Alps. ACT = Australian Capital Territory.

Within the state of Victoria, there are 79 defined local governmental areas and several “unincorporated” areas.^12^ The Australian Alps are the highest mountain range in Australia, spanning the states of New South Wales, Victoria and the Australian Capital Territory (Table 1 and Figure 1). Local governmental areas defined as alpine were Falls Creek Alpine Resort, Falls Creek Alpine Resort Unincorporated, Mount Baw Baw Alpine Resort, Mount Baw Baw Alpine Resort Unincorporated, Mount Buller Alpine Resort, Mount Buller Alpine Resort Unincorporated, Mount Hotham Alpine Resort, Mount Hotham Alpine Resort Unincorporated, or Mount Stirling Alpine Resort. To qualify as an alpine OHCA, the OHCA also had to occur during the months of June to September, which broadly correlate to the Australian ski season.

**Table 1:** The Victorian component of the Australian Alps

	Mount Buller Alpine Resort	Mount Hotham Alpine Resort	Falls Creek Alpine Resort
Elevation, m	1600	1861	1600
Visits in 2022	452,439	212,891	195,953
On-site medical services?	• Ambulance Victoria base on mountain • Oversnow ambulance on mountain • Retrieval via helicopter possible	• Oversnow ambulance on mountain • Retrieval via helicopter possible • Air ambulance (fixed-wing retrieval) via nearby Mount Hotham Airport	• Oversnow ambulance on mountain

### Historic visitation data

Alpine Resorts Victoria encompasses all defined alpine resorts within the state of Victoria and reports historic visitation data from 2007 to 2022. Visitation data were compiled by summing annual totals and modeling the pre-2007 years as a product of the median rates in 2007 to 2011. Each visit recorded equates to a day of alpine activity. Prior to 2015, this was calculated by purchase of a daily lift ticket, and assumptions made about the frequency of activity of seasonal lift-pass holders. From 2015 onward, numbers are more exact due to electronic lift ticket scanning capturing all ski field days accurately.

### Alpine emergency medical response

The primary response to cardiac arrest in alpine settings will typically be a ski patroller. Ski patrollers are paid first responders who are credentialled to an advanced first aid level by the Australian Ski Patrol Association (although many patrollers will have a background as paramedics, nurses or physicians). All ski patrollers have access to automatic external defibrillators (AEDs) and basic resuscitation equipment. Ambulance Victoria provides extra paramedic staffing to alpine areas in the ski season. These paramedics have all undertaken extra training in alpine response, and some have completed more comprehensive training in wilderness response. They will respond using specially equipped snowmobiles or all-terrain ambulance vehicles with self-laying tracks. All paramedics are equipped with defibrillators and advanced resuscitation equipment. Evacuation of successfully resuscitated patients will typically be via rotary wing transport to a tertiary center with percutaneous coronary intervention capability. Ambulance Victoria helicopters are crewed by specialist critical care paramedics with an advanced scope of practice.

### Inclusion criteria

OHCAs of presumed cardiac cause occurring between 2002 and 2021 attended by EMS were included in the study.

### Exclusion criteria

OHCA cases defined by the attending paramedic as occurring due to trauma, hanging, overdose/poisoning, terminal illness, or respiratory or other noncardiac causes were excluded from analysis. Cases who did not receive any attempt at resuscitation (typically due to clear absence of signs of life) and EMS-witnessed OHCAs were also excluded from the study, as characteristics of both these groups confound assessment of bystander interventions.

### Statistical analysis

Patients with alpine OHCA were compared with those with OHCA in a nonalpine environment occurring in a public setting. Within the cohort of patients with alpine OHCA, a descriptive analysis of circumstances and underlying OHCA precipitant was undertaken.

Categorical variables were assessed using a chi-square test, with results reported as absolute values and percentages. For continuous variables, normality was assessed using a Shapiro-Wilk test and statistical comparisons made using the Mann-Whitney test, with results reported as median (interquartile range).

A *P* value of <.05 was considered significant for all comparisons. All statistical analysis was performed using STATA/MP v17.0 (StataCorp).

### Ethical approval

VACAR holds overarching ethical approval from Monash University Human Research Ethics Committee, and the use of NCIS data is approved by the Justice Human Research Ethics Committee (CF/23/26172). These registries provide a waiver of consent for patient data inclusion according to the National Health and Medical Research Council of Australia requirements for providing a waiver of consent,^13^ ensuring that research adheres to relevant ethical guidelines. Of note, NCIS requirements mandate that cell values <5 are reported as “<5" or descriptively only, to preserve confidentiality. Conversely, where percentages allow unmasking of underlying numbers <5, these must be reported as “not provided.”

## Results

**Figure 2:**
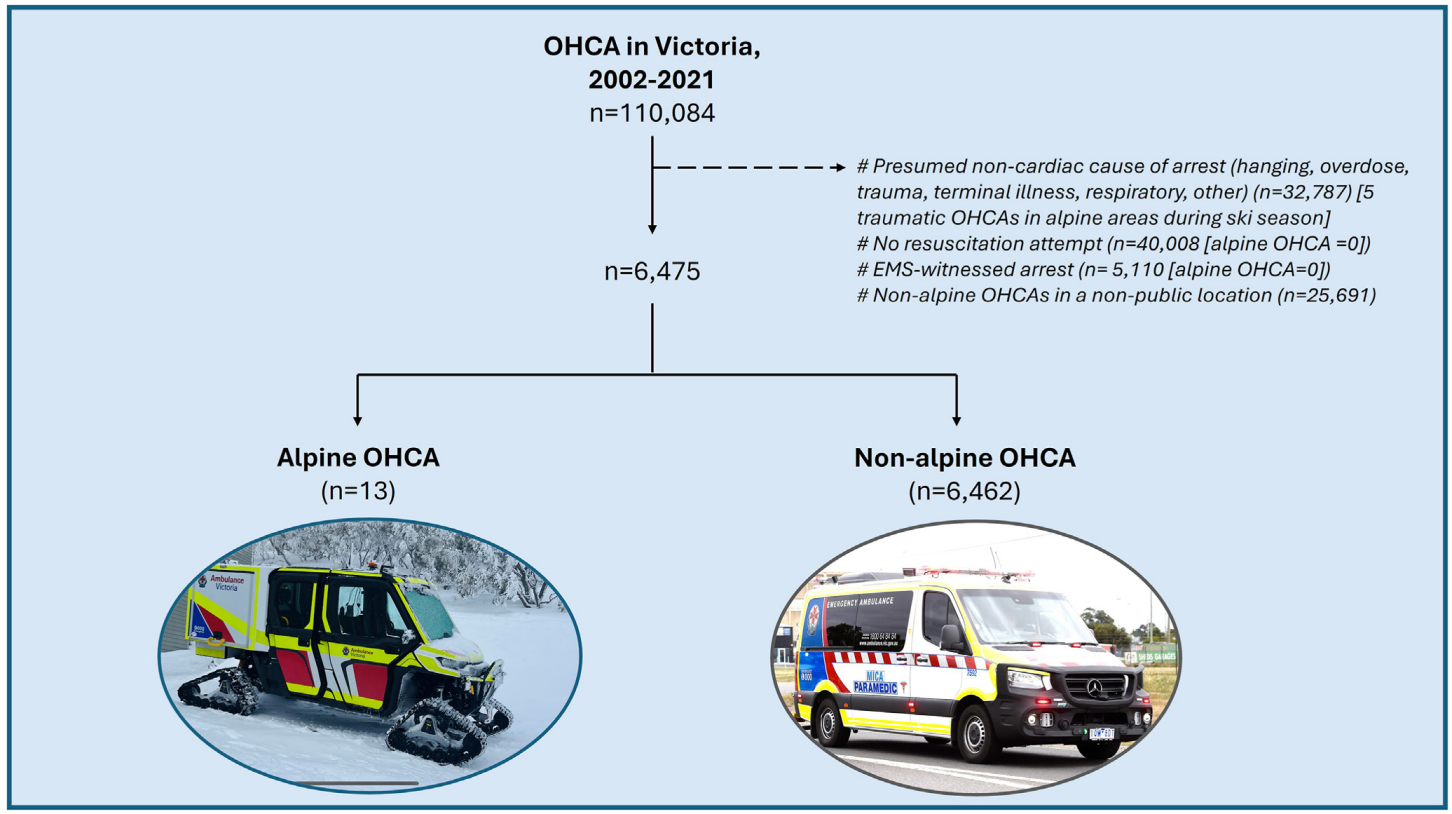
Flow diagram of patient identification and inclusion. EMS = emergency medical services; OHCA = out-of-hospital cardiac arrest.

Over the specified time period, approximately 15,277,000 alpine tourists were recorded at the Victorian alpine resorts. From a cohort of 32,179 OHCAs eligible for inclusion over the same time frame, 13 alpine OHCAs were identified (0.04% of OHCAs, 0.0001% of alpine visits) (Figure 2) and 6462 nonalpine OHCAs occurred in a public setting.

### Alpine vs nonalpine OHCA

The alpine OHCA cohort exhibited marked differences to the nonalpine OHCA cohort. Alpine OHCA patients were younger (median age 52 years vs 63 years, *P =* .0373) and very frequently male (>80%, partially masked number as per NCIS data requirements), although gender differences did not reach statistical significance. More favorable resuscitation parameters in the alpine cohort included higher rates of bystander defibrillation using a publicly accessible defibrillator (54.5% vs 13.5%, *P <* .0001) (Table 2).

**Table 2:** Clinical profiles and outcomes of alpine vs nonalpine OHCA

	Alpine OHCA (n = 13)	Nonalpine OHCA (n = 6462)	Significance
Age, y	52 (45–64)	63 (52–74)	.0373∗
Male	n.p. (>90)†	5446 (84.3)	.428
Time to ambulance arrival, min	13.7 (2–34.4)	8.1 (6–11)	.2721
Witnessed arrest	8 (61.5)	4957 (77.2)	.180
Bystander CPR	12 (92.3)	4724 (73.1)	.119
Initial rhythm VF/VT	11 (84.6)	4260 (66.3)	.163
Bystander defibrillation	6 (54.5)	661 (13.5)	<.0001∗
Outcomes			
Survived to hospital	7 (53.8)	3008 (46.9)	.427
Survived to discharge	5 (38.5)	1827 (28.8)	.323

Values are median (interquartile range) or n (%).

CPR = cardiopulmonary resuscitation; OHCA = out-of-hospital cardiac arrest; VF = ventricular fibrillation; VT = ventricular tachycardia.

∗ Significant at *P* < .05 level.

† Exact number not provided due to potential identifiability.

Survival to hospital discharge did not differ between cohorts (38.5% vs 28.8% for all initial rhythms, *P =* .323).

### Causes of OHCA

The 13 cases of alpine OHCA were examined; 7 OHCAs (38.5%) occurred on or near the ski slope, including a small number on the chairlift. A number of these cases received bystander defibrillation on-slope. Six OHCAs occurred at residential areas such as ski lodges, with the patient in a sedentary state.

Eight patients were transferred to major tertiary centers in metropolitan Melbourne for postarrest care, with 5 (38.5%) patients surviving to discharge, for whom the most common etiology of OHCA was an acute coronary syndrome. Of the 8 patients who did not survive (5 deceased prehospital), 6 (75.0%) proceeded to autopsy examination. The commonest cause of OHCA found at autopsy was acute coronary syndrome, and the majority of patients had pre-existing cardiovascular risk factors including obesity, diabetes, cardiomyopathy, and documented previous myocardial infarction.

## Discussion

Our study demonstrates that, while previous studies have reported that SCA is a major cause of alpine mortality, the absolute risk of alpine SCA is very low at approximately 1 per million ski field visits. Prognostically positive patient characteristics and resuscitation factors were observed, with almost 40% of alpine OHCA patients surviving to hospital discharge.

### Rates of alpine OHCA

In Table 3, we present an overview of prior studies on alpine OHCA, predominantly from the European Alps. As can be seen, a methodological strength of our study is the inclusion of both a comparator group and underlying information on OHCA etiology. In 2007, Burtscher^7^ reported from Austria that OHCA was the number one cause of mortality in males over 34 years old in the mountains. Interestingly, 50% of all sudden cardiac deaths occurred on the first day spent at altitude; we did not have equivalent data in our study to be able to interrogate this pattern. Although this statistic presented an alarming image of alpine OHCA, incidence and exposure data were unable to be calculated; thus, the overall risk profile was incomplete.

**Table 3:** Previous studies on alpine OHCA

Author, year, country	Number of alpine OHCAs	Number in comparator group	Age (y)	Male (%)	OHCA etiology	Comments
Sherry and Clout, 1988, Australia^15^	15	—	53 (median)	93.3	Predominantly acute coronary syndromes	No comparator group
Eisenburger et al, 2001, Austria^26^	368	—	64 (median)	69	n.p.	No comparator group
Burtscher, 2007, Austria^7^	247	741	>60 (required to be >34 for inclusion) (median)	93.7	n.p.	Comparator cohort did not experience OHCA, but were hikers matched on age and gender
Chacornac et al, 2010, France^21^	9	—	n.p.	n.p.	Coronary as all STEMI patients	Descriptive analysis of STEMI patients, specific demographics of OHCA patients not provided No comparator group for STEMI patients
Hållmarker et al, 2012, Sweden^17^	20	—	55 (mean)	100	Predominantly acute coronary syndromes	No comparator group
Ruedl et al, 2011, Austria^16^	79	—	60.5	93.5	n.p.	No comparator group
Viglino et al, 2017, France^22^	136	12,500	56	89	n.p.	Comparator group was off-slope events but not required to be in a public setting
Strohle et al, 2019, Austria^23^	785	—	60	92	n.p.	No comparator group
Paratz et al, 2025, Australia (current study)	13	6462	52	>90	Predominantly acute coronary syndromes	—

n.p. = not provided; OHCA = out-of-hospital cardiac arrest; STEMI = ST-segment elevation myocardial infarction.

Our findings of only 0.8 OHCAs per million ski days accord with earlier studies demonstrating a low absolute risk of alpine OHCA. The only other Australian alpine study dates from 1988, when Sherry and Clout analyzed data from the New South Wales component of the Australian Alps and calculated a rate of 0.45 OHCAs per million skier days.^14^^,^^15^ This is approximately half the rate identified in our study, which may provide support for the theory that alpine tourist age and medical comorbidities are increasing. In Austria, Ruedl and colleagues^16^ calculated all-cause mortality to be 0.8 deaths per million skier days, of which 73% were due to cardiac arrest; a roughly similar rate to our study. As presented in Table 1, altitudes of the European and Australian alps are largely comparable.

In the Swedish Vasaloppet ski race, Hållmarker and colleagues^17^ reported a 21.6 per million event rate for OHCA; notably, this is a high-intensity 90-km ski race, which is likely to differ substantially from a typical day of recreational alpine activity captured in our study and others. Downhill skiing is estimated to expend 4.3 to 12.5 metabolic equivalents, while cross-country skiing is estimated to expend 6.8 to 16.0 metabolic equivalents, reflecting a substantial difference on energy expenditure.^18^ Interestingly, causes of OHCA in the Vasaloppet ski race were universally cardiac, comprising coronary artery disease, hypertrophic cardiomyopathy, myocarditis, and idiopathic ventricular fibrillation.^17^ This differs from the usual pattern, in which approximately 45% of OHCAs are subsequently determined to have a noncardiac etiology.^19^^,^^20^

### Patient characteristics

Alpine OHCA patients in our study exhibited differences from nonalpine OHCA patients, being younger and receiving higher rates of bystander defibrillation. These baseline differences in alpine OHCA appear consistent across multiple studies. A French analysis of 114 ST-segment elevation myocardial infarction cases in ski resorts of the French Alps demonstrated a similar overwhelming male proportion (93%) of cases and relatively young mean age of just 57 years.^21^ Another French study, which focused on alpine OHCA over a 10-year period, again demonstrated that males comprised 89% of OHCAs, with alpine OHCA patients having a median age of 56 years and being more commonly identified to be in an initial shockable rhythm.^22^

One group that has differed from our findings is the Austrian cohort, who have reported survival rates much lower than ours (0.9% vs 39%). This is likely driven by markedly lower rates of bystander interventions in the Austrian Alps, with only a 17% rate of bystander cardiopulmonary resuscitation (CPR) compared with 93% in our cohort.^23^ High rates of witnessed OHCA and bystander interventions identified in this study were strongly predictive of survival. All ski patrollers and ski instructors on Australian mountains must hold first aid certification, which increases the density of people capable and able to provide bystander interventions, translating into excellent outcomes in this study.^24^

### Resuscitation in an alpine environment

In 2005, Lienhart and colleagues^25^ stated that “in Alpine regions, optimal survival times for professional rescue services are very difficult to achieve….in high alpine regions, complete adherence to the rescue chain is a prerequisite for the survival of those affected.” In this study, it was a reassuring finding that time for EMS arrival did not differ between alpine and nonalpine OHCA.

As in our study, Eisenburger and colleagues^26^ reported in Austria over a 6-year time period in an alpine area that EMS response times and the OHCA survival rate were equivalent to metropolitan values. This was, however, in an area with permanent EMS staffing, 16 ambulances, and 3 helicopters available to reach all parts of the mountain within 15 minutes during daytime hours. This degree of resourcing differs markedly from the typical Australian alpine environment.^26^ In the French setting, time from first call to arrival of first responders was an average of 11 minutes, with no difference seen between on- and off-slope events.^22^

Multiple study groups have suggested that mechanical CPR devices may be particularly useful when patients require extrication off a ski slope or there are extraction difficulties such as winching to a helicopter, as they may enable ongoing CPR where it would not otherwise be possible.^27^^,^^28^ Perhaps unsurprisingly, CPR in a moving toboggan has been objectively confirmed on videometric analysis to be of lower quality than CPR on a stationary patient.^29^ The number of such cases would be anticipated to be limited, but it could be feasible to consider a per-mountain purchase to be directed toward cases anticipated to have difficulties in providing field CPR.^30^ It has also been noted that performing prolonged CPR at relative hypoxia is challenging for rescuers, leading to poorer-quality efforts and earlier fatigue.^31^^,^^32^

The excellent survival rates in Australian alpine OHCA appear to have occurred despite, not as a result of, the challenges of the alpine environment. As such, it is important to maximize the prognostically favorable elements of alpine resuscitation such as high rates of bystander intervention and early access to defibrillation.

### Future directions

Future initiatives to improve alpine OHCA outcomes encompass both the organizational and individual levels. On an organizational level, early bystander defibrillation contributed demonstrably to improved survival in remote alpine regions in this study, and efforts to increase defibrillator density in alpine resorts should be further encouraged. In 2006, official guidelines were issued from the International Commission for Mountain Emergency Medicine, recommending that public access defibrillators should be placed with priority in popular ski areas, in busy mountain huts and restaurants, at mass participation events, and in remote but often visited locations that do not have medical coverage.^33^ Ongoing training of all ski patrol in defibrillator use remains of high importance.

On a personal level, consensus recommendations in a range of cardiovascular conditions and risk factors have previously been published for people with cardiac comorbidities wishing to undertake alpine sports.^9^ In the context of an aging population and the established risk of cardiovascular risk factors in alpine SCA, clinicians are likely to manage a large cohort of patients who intend to ski. Given the 92.9-fold elevated risk of SCA in people with a previous myocardial infarction and 9-fold increased risk in people with hypertension,^34^ it would be reasonable to advise people identified as high risk not to ski alone. In an era of ultraportable AEDs, it may also be feasible for high-risk skiiers to carry their own AEDs as a protective measure (noting that currently only one brand of ultraportable AED is licensed for use at temperatures below 0 °C).^35^ While carrying ultraportable AEDs would be highly unlikely to be cost-effective on a broader level, it may be an individual choice that some skiers wish to consider. Such measures may help further improve outcomes for alpine OHCA and support ongoing engagement throughout life in a range of winter sports.

### Limitations

The absolute number of alpine OHCAs in this study is small. There is a perception that Australia is not a classically alpine country; indeed, our data indicate the Victorian Alps see approximately 1% of global alpine visitation. However, we present 20 years of statewide data encompassing more than 15 million alpine visits, resulting in a comprehensive dataset that will still inform clinical practice. It is worth noting that we excluded cases of traumatic alpine OHCA (n = 5), which would also require resuscitation resources similar to presumed cardiac OHCA in the prehospital setting. We also excluded cases of OHCA in the same geographic location occurring outside winter months (n = 6), who would have experienced some of the same access issues.

## Conclusion

Alpine OHCA is very rare in Australia, accounting for 1 in 2500 OHCAs and affecting slightly fewer than 1 in a million ski field visitors. Ischemic heart disease is the commonest etiology, often occurring on a background of known risk factors or established coronary disease. Alpine OHCA patients are almost 20 years younger than the typical Australian OHCA, and due to higher rates of bystander CPR, shockable rhythms, and bystander defibrillation, their survival is higher than nonalpine OHCA, driven by positive baseline characteristics and resuscitation factors.
